# Prognostic value of serum albumin-to-creatinine ratio in patients with acute myocardial infarction: Results from the retrospective evaluation of acute chest pain study

**DOI:** 10.1097/MD.0000000000022049

**Published:** 2020-08-28

**Authors:** Hong Liu, Jianna Zhang, Jing Yu, Dongze Li, Yu Jia, Yisong Cheng, Qin Zhang, Xiaoyang Liao, Yanmei Liu, Jiang Wu, Zhi Zeng, Yu Cao, Rui Zeng, Zhi Wan, Yongli Gao

**Affiliations:** a Department of Emergency Medicine, Laboratory of Emergency Medicine, Deep Underground Space Medical Center, West China School of Nursing, West China Hospital, and Disaster Medical Center, Sichuan University, Chengdu, China; b West China School of Nursing, West China Hospital, Sichuan University, Chengdu, China; c Department of General Practice, International Hospital of Sichuan Province, National Clinical Research Center for Geriatrics, West China Hospital, Sichuan University, Chengdu, China; d Chinese Evidence-based Medicine Center, West China Hospital, Sichuan University, Chengdu, China; e Department of Cardiology, West China Hospital, Sichuan University, Chengdu, China.

**Keywords:** acute myocardial infarction, biomarker, mortality, prognosis, serum albumin-to-creatinine ratio

## Abstract

The long-term association between serum albumin-to-creatinine ratio (sACR) and poor patient outcomes in acute myocardial infarction (AMI) remains unclear. This study aimed to determine whether sACR was a predictor of poor long-term survival in patients with AMI.

This was a study of patients with AMI in the emergency department (ED) from the retrospective multicenter study for early evaluation of acute chest pain (REACP) study. The patients were categorized into tertiles (T1, T2, and T3) based on the admission sACR (0.445 and 0.584 g/μmol). Baseline sACR at admission to the ED was predictive of adverse outcomes. The primary outcome was all-cause mortality within the follow-up period. Cox proportional hazards models were performed to investigate the association between sACR and all-cause mortality in patients with AMI.

A total of 2250 patients with AMI were enrolled, of whom 229 (10.2%) died within the median follow-up period of 10.7 (7.2–14.6) months. Patients with a lower sACR had higher all-cause mortality and adverse outcomes rates than patients with a higher sACR. Kaplan–Meier survival analysis showed that patients with a higher sACR had a higher cumulative survival rate (*P* < .001). Cox regression analysis showed that a decreased sACR was an independent predictor of all-cause mortality [T2 vs T1: hazard ratio (HR); 0.550, 95% confidence interval (95% CI), 0.348–0.867; *P* = .010 and T3 vs T1: HR, 0.305; 95% CI, 0.165–0.561; *P* < .001] and cardiac mortality (T2 vs T1: HR, 0.536; 95% CI, 0.332–0.866; *P* = .011 and T3 vs T1: HR, 0.309; 95% CI, 0.164–0.582, *P* < .001).

The sACR at admission to ED was independently associated with adverse outcomes, indicating that baseline sACR was a useful biomarker to identify high-risk patients with AMI at an early phase in ED.

## 1 Introduction

Acute myocardial infarction (AMI) is an emergent form of coronary artery disease and is associated with high mortality.^[1]^ Although the mortality rate of patients with AMI has decreased with advances in medical and interventional treatment, ischemic heart disease remains a major cause of death in China.^[2]^ Prompt treatment of AMI improves the survival rate, and risk assessment of patients with AMI at the time of admission to the emergency department (ED) helps to guide clinical decision-making and improve the effectiveness of treatment.^[1,2]^ Among the current risk stratification tools, the Global registry of Acute Coronary Events (GRACE) and Thrombolysis in Myocardial Infarction (TIMI) risk scores are widely used for prognostic assessment. They include medical history, biochemical variables, markers of myocardial injury, and electrocardiography findings.^[1,3,4]^ Results of previous studies indicated multiple pathophysiological biomarkers that have prognostic value for the evaluation of patients with cardiovascular diseases.^[5–7]^ Some simple biomarkers may have greater prognostic value for patients with early-phase AMI in the ED than complex risk scores.

AMI is associated with inflammatory responses and thrombosis accompanied by a significant elevation in the levels of inflammatory markers, including D-dimer and C-reactive protein (CRP).^[5,8,9]^ However, results of other studies have indicated that elevation in CRP or D-dimer levels at admission is not associated with adverse outcomes in patients with AMI.^[8,9]^ The thrombo-inflammatory status may reflect the severity of cardiovascular diseases, and a combination of an inflammatory and a thrombotic biomarker may be more predictive of outcomes than a single inflammatory or thrombotic biomarker; however, complex calculations would still be required.^[5]^ Serum albumin (SA) is a stable protein associated with inflammation and platelet activation and was found to be an important independent biomarker for adverse outcomes of AMI.^[10,11]^ Acute kidney injury (AKI) is a common target organ injury and is reported to be associated with a poor prognosis in patients with AMI.^[12]^ Serum creatinine (sCr), a biomarker of AKI, was found to be associated with increased risks of mortality in patients with AMI.^[13]^ The urine albumin-to-creatinine ratio (uACR) has been recommended as a suitable marker for monitoring proteinuria and is independently associated with increased long-term risks of cardiovascular and total mortality in survivors of myocardial infarction.^[14]^ Therefore, we hypothesize that the sACR will provide additional prognostic information at early-phase AMI in the ED. To verify this, we conducted this retrospective multicenter cohort study to investigate the usefulness of the baseline sACR to identify high-risk patients with AMI on admission to the ED.

## 2 Materials and methods

### 2.1 Study design

We analyzed data from the multicenter retrospective evaluation of acute chest pain (REACP) study. The REACP study included patients with acute chest pain on admission to the ED of acute chest pain centers at seven tertiary hospitals in China between January 2017 and December 2018. The REACP study is registered at www.chictr.org.cn (Identifier: ChiCTR1900024657). In the present study, we evaluated whether the sACR predicted mortality and adverse outcomes in patients with AMI undergoing primary percutaneous intervention (PCI) in the ED. The study was conducted in accordance with the Declaration of Helsinki and was approved by the Institutional Review Boards of Sichuan University West China Hospital and other participating hospitals.

### 2.2 Study population

**Figure 1:**
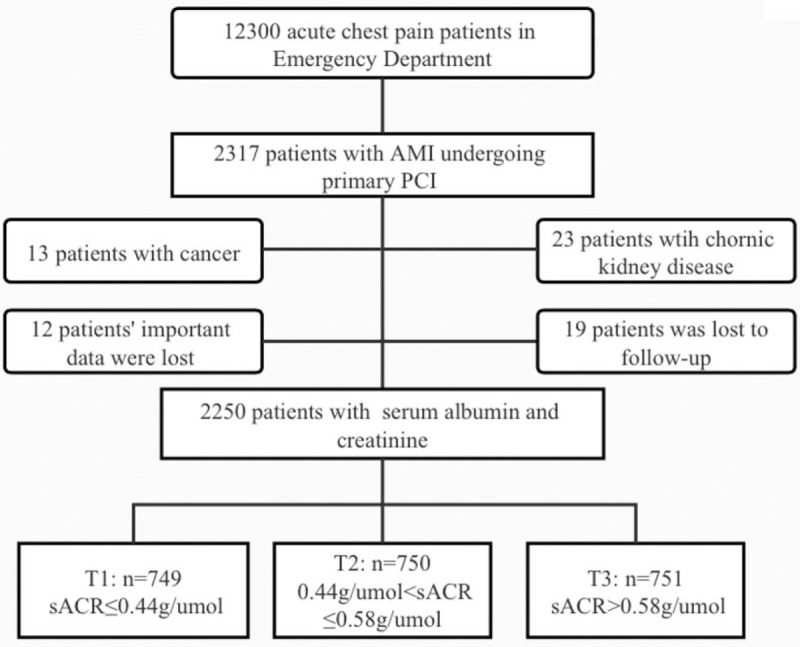
Study flow chart. AMI = acute myocardial infarction, PCI = percutaneous coronary intervention, sACR = serum albumin-to-creatinine ratio.

From January 2017 to December 2018, a total of 2250 of the 12,300 patients with acute chest pain with AMI who were enrolled in the REACP study and underwent primary PCI in the ED were included in this study. The inclusion criteria were as follows: age >18 years, first-time diagnosis of ST-elevation myocardial infarction or non-ST-elevation myocardial infarction, <12 hours between the onset of symptoms and ED admission, and treated with coronary angiography and primary PCI. The exclusion criteria were as follows: presence of malignant tumors, chronic systolic heart failure, history of chronic hepatopathy or chronic renal disease, and loss to follow-up. Chronic systolic heart failure was defined as a clinical syndrome characterized by typical symptoms (e.g., breathlessness, ankle swelling, and fatigue) with reduced left ventricular ejection (considered as <40%). Chronic renal disease was defined as the presence of either of the conditions listed below lasting for more than 3 months, including kidney damage: abnormal findings in blood or urinary tests, imaging studies, or pathological evaluations and estimated glomerular filtration rate (eGFR) <60 mL/min/1.73 m^2^. Figure 1 is a diagram of patient selection.

### 2.3 Data collection

Baseline data on patient demographic and clinical characteristics such as vital signs, medical histories, laboratory tests, electrocardiograms, cardiac color Doppler ultrasound, coronary angiography, in-hospital complications, and treatment before admission obtained during the hospital stay and at discharge were collected from the electronic medical records at each participating hospital. The sACR was calculated based on assays performed to assess SA and sCr levels on admission to the ED. Laboratory tests were performed using standard procedures of the Sichuan University West China Hospital. Patients with a body mass index (BMI) of >24 kg/m^2^ were classified as overweight.

### 2.4 Endpoint and follow-up

The primary endpoint was all-cause mortality, and the secondary endpoint was cardiac death that was diagnosed as death caused by myocardial infarction, cardiac arrest, heart failure, life-threatening arrhythmias, and other cardiac events. All reported events were reviewed and validated by an outcome assessment committee that was blinded to the treatment assignment. The follow-up period was calculated from the onset date of AMI to the date of an event or the date of the last follow-up. In-hospital and post-discharge outcomes were collected from medical records, during hospital visits, or by telephone interviews.

### 2.5 Statistical analysis

The patients were categorized into tertiles (T1, T2, and T3) based on the admission sACR (0.445 and 0.584 g/μmol). Continuous variables were reported as means ± standard deviation or medians (25th and 75th percentile). Categorical variables were reported as numbers and percentages. Parametric patient characteristics were compared using 1-way analysis of variance. Nonparametric characteristics were compared using the Kruskal–Wallis H test. Categorical data were compared using Chi-square tests. Cumulative survival of patients in the 3 sACR tertiles was estimated using the Kaplan–Meier method and log-rank tests, and Cox proportional hazards models were used to identify the associations between the sACR categories and survival. Hazard ratios (HRs) and 95% confidence intervals (CIs) were calculated for the 2 higher sACR tertiles, with the lowest tertile serving as a reference. Cox proportional hazards models were performed using age, sex, hypertension, Killip class, type 2 diabetes mellitus (T2DM), troponin T (TnT), left ventricular ejection fraction (LVEF), and GRACE and Gensini scores. Receiver operating characteristic (ROC) curves, continuous net reclassification index (NRI), and integrated discrimination improvement were performed to evaluate improvement in prognosis following addition of the sACR to the GRACE score in the statistical models.^[15]^ Subgroup analysis of Cox proportional hazards models was performed to test the robustness of the association between the sACR and the primary endpoint after adjusting for covariates unless the variable was used as a subgroup variable.

The significance level was a 2-tailed *P*-value of <.05. Statistical analysis was performed using SPSS version 20.0 (IBM Corp, Armonk, NY) and Stata version 14.0 (Stata Corp, College Station, TX).

## 3 Results

### 3.1 Baseline patient characteristics

In total, 2250 patients with AMI with an average age of 64.8 ± 13.1 years were included; of these patients, 1698 (75.5%) were men, and the median follow-up was 10.7 (7.2–14.6) months. A total of 229 (10.2%) patients died, and the causes of death were cardiac death in 214 (93.4%), stroke in 4 (1.7%), inflammation in 8 (3.5%), bleeding in bleed (0.4%), and accidents in 2 (0.9%). Patients in group T1 were older; had lower BMIs, systolic blood pressure (SBP), diastolic blood pressure (DBP), LVEFs; hemoglobin, albumin, eGFR, triglyceride, total cholesterol, high-density lipoprotein, and low-density lipoprotein levels; lower sACR and incidence of left anterior descending artery infarction; this group also had more patients with hypertension; T2DM; high heart rate; high Killip class; high white blood cell (WBC) count; high levels of fibrinogen, D-dimer, random blood glucose, sCr, blood urea nitrogen (BUN), N-terminal pro-brain natriuretic peptide (NT-proBNP), and troponin T; and high left main and multiple-vessel stenosis and baseline GRACE and Gensini scores compared with those in the higher sACR tertile groups (Table 1).

**Table 1:**
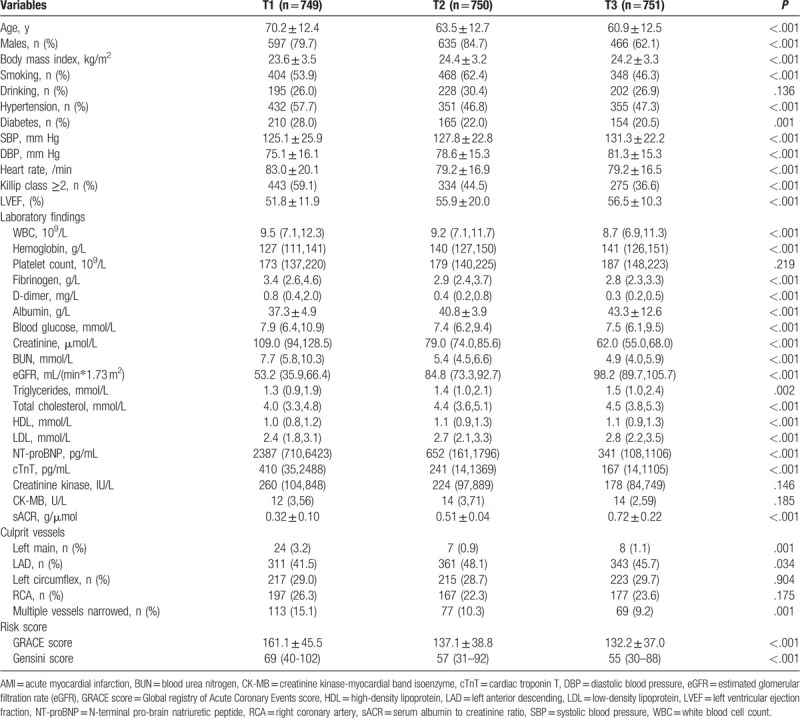
Baseline clinical characteristics of sACR groups in AMI patients.

### 3.2 sACR level and clinical outcome

**Figure 2:**
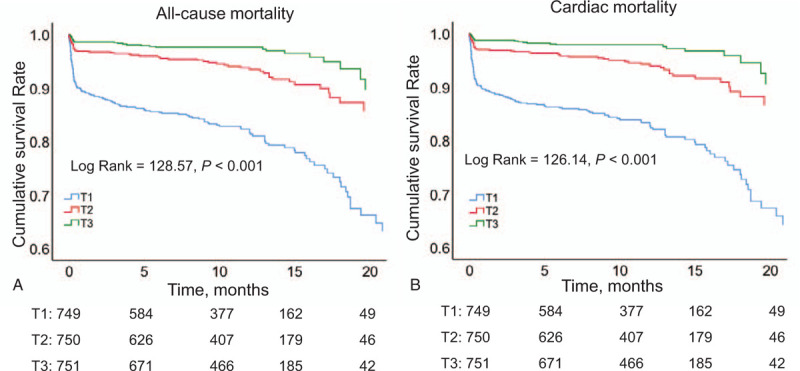
Kaplan–Meier survival curve of all-cause death and cardiac death for AMI patients by sACR. AUC = area under curve, CI = confidence interval, cTnT = cardiac troponin T.

Patients with a high sACR had lower in-hospital risks of all-cause mortality, cardiac death, and other adverse outcomes than those with a low sACR. During the follow-up period, patients with a low sACR had increased all-cause mortality, cardiac death, and other adverse outcomes compared with those with a high sACR (Table 2). Kaplan–Meier analysis revealed that patients with a low sACR had poorer cumulative survival than those with a high sACR regardless of all-cause and cardiac mortality (Fig. 2, *P* < .001 for both). After adjusting for confounding factors, Cox regression analysis revealed that a decreased sACR was an independent predictor of all-cause mortality (T2 vs T1: HR, 0.550; 95% CI, 0.348–0.867; *P* = .010 and T3 vs T1: HR, 0.305; 95% CI, 0.165–0.561; *P* < .001) and cardiac mortality (T2 vs T1: HR, 0.536; 95% CI, 0.332–0.866; *P* = .011 and T3 vs T1: HR, 0.309; 95% CI, 0.164–0.582; *P* < .001; Table 3).

**Table 2:**
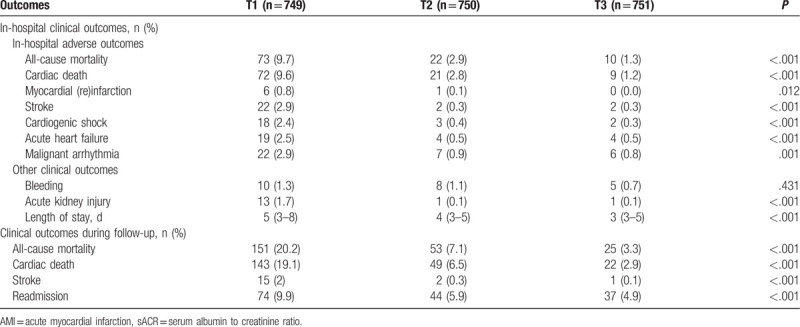
Clinical adverse outcomes of sACR groups in AMI patients.

**Table 3:**
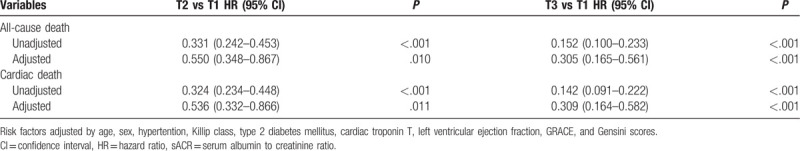
Cox regression analysis regarding correlations between clinical outcomes and sACR.

### 3.3 Predictive value of sACR

**Figure 3:**
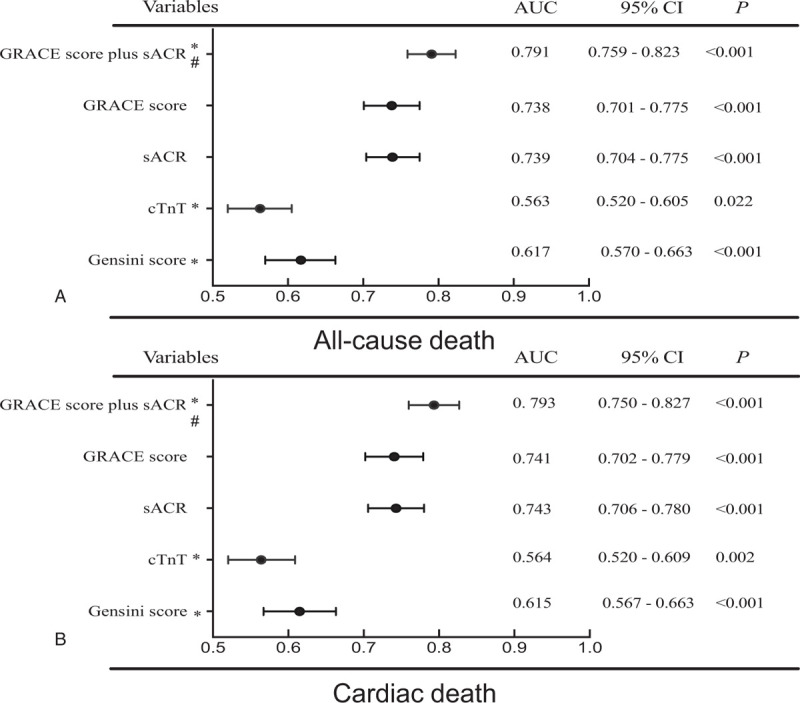
Area under the receiver operating characteristic curve of the sACR and other risk factors and scores. GRACE score = Global registry of Acute Coronary Events score, sACR = serum albumin-to-creatinine ratio, sACR = serum albumin-to-creatinine ratio. ^∗^Compared with AUC of sACR, the difference is significant (*P* < .05). ^†^Compared GRACE score and sACR with GRACE score of AUC is significant (*P* < .05).

The area under the curve (AUC) generated by ROC curve analysis showed that the sACR was superior to both the Gensini score and cardiac troponin T (cTnT) in predicting all-cause mortality (Fig. 3A, both *P* < .05). The AUCs of all-cause mortality for the sACR and GRACE score did not differ significantly (*P* > .05). ROC curve analysis revealed that combining the sACR and GRACE score was more predictive of mortality than the GRACE score alone (AUC, 0.791 vs 0.738; *P* = .002). Using 10% and 15% as arbitrary thresholds to define patients at low, medium, and high risk of mortality, the sACR achieved an NRI of 0.14 (95% CI, 0.07–0.20; *P* < .001; Table 4). ROC curve analysis also showed that the sACR was superior to the Gensini score and cTnT and similar to the GRACE score in predicting cardiac mortality. However, the sACR improved the prognostic value of the GRACE score (Fig. 3B).

**Table 4:**
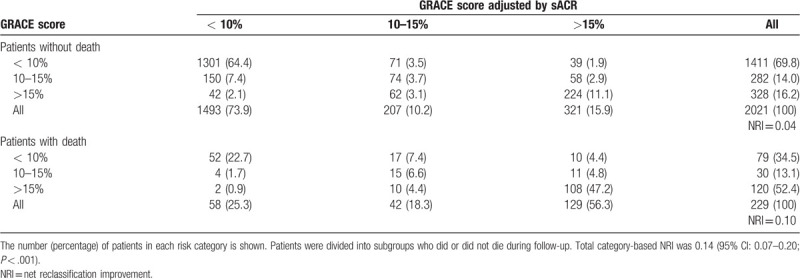
Reclassification across pre-defined risk thresholds in the validation cohort using the algorithm for GRACE score adjustment by sACR developed in the derivation cohort.

### 3.4 Subgroup analysis

Cox regression analysis revealed that a decreased sACR was independently associated with all-cause mortality in patients with AMI in subgroups based on different levels of sex, age, BMI, SBP, DBP, heart rate, WBC, platelet count, hemoglobin, cTnT, NT-proBNP, urine protein, Killip class, GRACE score, and AMI type (Table 5).

**Table 5:**
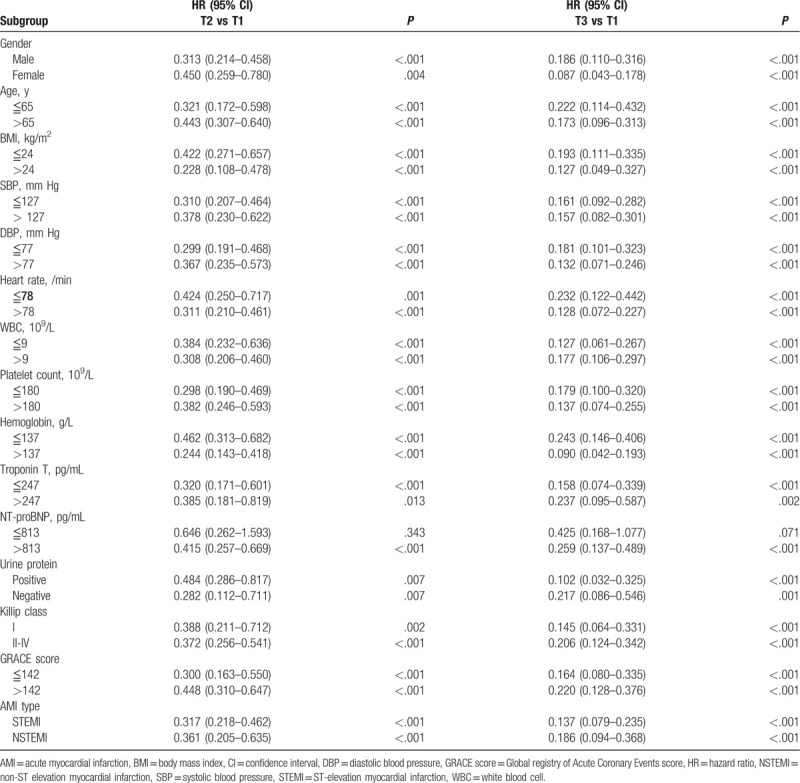
Kaplan–Meier survival analysis of mortality in AMI patients.

## 4 Discussion

In this study, we evaluated the prognostic value of sACR in predicting adverse outcomes in patients with AMI undergoing PCI. A low sACR was associated with increased risks of all-cause mortality and adverse outcomes compared with a high sACR, with a gradual dose–response association. A low sACR independently predicted a poor prognosis. The sACR improved the prognostic value of the GRACE score for stratifying high-risk patients with AMI on admission to the ED. The sACR was shown to be useful as a biomarker for risk stratification of patients with early-phase AMI in the ED.

Inflammation and thrombosis are involved in the occurrence and development of AMI.^[16]^ Results of previous studies have found that the thrombo-inflammatory status reflects the severity of AMI and that a thrombo-inflammatory prognostic system is more predictive than a single inflammatory or thrombotic marker.^[5,6,17]^ SA is a negatively charged plasma protein with antioxidant functions that can signal inflammatory regulatory changes in cells that is dependent on their redox state.^[18–20]^ Results of previous studies revealed that decreased synthesis and increased catabolism of SA were associated with inflammation and that SA inhibited platelet activation and aggregation and was a mediator of platelet-induced AMI^[21,22]^ and can predict adverse outcomes of patients with AMI.

Hypoalbuminemia has been associated with an increased risk of both in-hospital and long-term adverse outcomes in patients with acute aortic dissection or AMI admitted to the ED.^[5–7,10,11,23]^ Decreased SA levels have also been associated with the development of cardiac insufficiency in patients with AMI who undergo primary PCI, and a continuing decrease in SA levels in patients with heart failure is correlated with poor prognosis. In a previous study, SA level was particularly noteworthy as an indicator for frailty that was associated with adverse outcomes. Man et al^[24]^ reported a 2- to 5-fold higher risk of in-hospital, short-, and long-term mortality in elderly patients with acute coronary syndrome. Changes in SA levels have also been associated with changes in cachectic factors.^[25–27]^ SA levels together with scores of indexes such as the inflammation-based Glasgow prognostic score and prognostic nutritional index can be used to stratify high-risk patients with early-phase AMI in the ED.^[5,28]^ Finally, a reduction in the levels of SA to a normal level has been associated with an increased incidence of cardiovascular diseases.^[29]^ In this study, the SA level was associated with adverse outcomes, but the prognostic value was limited, and a more accurate biomarker is needed for early evaluation of patients with AMI in the ED.

Early multiple organ damage is a known predictor of in-hospital and long-term mortality in patients with AMI, and AKI commonly occurs in patients with AMI.^[30,31]^ Patients with AMI having AKI have a 3- to 5.28-fold higher mortality risk than those without AKI.^[30,32]^ sCr is a biomarker of AKI, and the initial sCr levels and daily changes in sCr levels are predictive of in-hospital mortality in patients with or without AMI.^[33,34]^ Elevated sCr levels have also been associated with peripheral endothelial function dysfunction, and an increase in the sCr levels within the normal range has been independently associated with cardiovascular disease risk in patients without metabolic syndrome.^[35,36]^ Therefore, sCr, a biomarker of AKI, might reflect the severity of AMI and facilitate the stratification of high-risk patients with early-phase AMI.

The uACR is associated with renal function damage, cardiovascular disease, subclinical hypothyroidism, etc.^[37]^ It reflects the pathophysiological changes that occur during AMI, reflecting both the severity of inflammation and degree of heart failure. Patients with higher uACR have a 3.6- to 4.9-fold increased risk of mortality.^[14]^ The sACR can be used as a simple marker of thrombo-inflammatory status and AKI.

In this study, we found that the sACR had a higher prognostic value than the SA and sCr levels and the TIMI score and that it had a gradual dose–response association with mortality. The ability of the sACR (AUC: 0.739) in predicting all-cause mortality was similar to that of the GRACE score (AUC: 0.738). However, determining the GRACE score involves the use of a computer or calculator to calculate the total score outcomes, which includes age, heart rate, SBP, Killip class, cardiac arrest, ST-segment deviation, and abnormal cardiac enzymes and is thus not suitable for use in the ED. Fortunately, the popularization of smartphone apps minimizes these drawbacks. On the contrary, the GRACE score does not include markers of pathophysiological status. Thus, the ability of the sACR to predict cardiac death is inferior to that of the GRACE score. Previous studies have reported that some potential inflammation-related risk factors could improve the predictive ability of the GRACE scoring system in terms of major adverse cardiac events or mortality.^[38]^ For the first time, we combined the sACR and GRACE score to predict adverse outcomes in patients with AMI; the results showed that the sACR could significantly improve the prognostic value of the GRACE score.

In addition to clinical factors, we identified differences between the genders in several demographic factors. The difference in mortality rates between women and men is currently disputed; most studies report higher mortality rates for women, whereas others report higher mortality rates for men or nonsignificant differences.^[39,40]^ After adjusting for age, the mortality rate between men and women was not significantly different. A previous study had demonstrated differences between the genders in the interaction of the risk scores on post-ACS prognosis such as Killip class and TIMI score.^[41]^ In the present study, the sACR was effective in predicting mortality for both women and men, but women had a higher risk of mortality than men in group T2 and a lower risk of mortality than men in group T3. This study focused on the correlation between the degree of sACR elevation and mortality. The specific differences between the genders need to further studied, with a focus on developing new specific scores. Other subgroup analyses indicated that the prognostic value of the sACR was stable in patients with different levels of cardiovascular risk factors. The sACR improved the prognostic value of widely used scoring systems for stratifying high-risk patients with early-phase AMI in the ED.

### 4.1 Limitations

This study has limitations. First, this was a retrospective study, and a large, prospective, multicenter study is needed to confirm the findings. Second, changes in the sACR during hospitalization and its association with adverse outcomes could not be investigated. Third, immune function, platelet activity, and thrombosis data to confirm the relationship between the sACR and inflammatory and thrombotic status could not be collected. Fourth, eGFR was not measured directly but was estimated. Finally, the time needed to determine the sACR after ED admission was unknown.

## 5 Conclusion

The sACR was found to be an independent prognostic marker in patients with AMI on admission to the ED regardless of the severity of AKI and thrombo-inflammatory status. When used in combination, the sACR improved the prognostic value of the GRACE score. The sACR is a useful marker for early risk stratification of patients with AMI.

## Author contributions

**Conceptualization:** Zhi Wan, Yongli Gao, Yu Cao and Zhi Zeng, Jianna Zhang.

**Data analysis:** Hong Liu, Jianna Zhang, Dongze Li, Jing Yu, Yu Jia, Yisong Cheng, Qin Zhang, Xiaoyang Liao and Zhi Wan.

**Data curation:** Jianna Zhang.

**Formal analysis:** Yanmei Liu and Hong Liu, Jianna Zhang.

**Methodology:** Yanmei Liu and Dongze Li.

**Project administration:** Zhi Wan, Rui Zeng, Yu Cao and Zhi Zeng.

**Supervision:** Rui Zeng, Zhi Wan and Zhi Zeng.

**Validation:** Zhi Wan, Rui Zeng and Yongli Gao.

**Writing – original draft:** Hong Liu and Dongze Li, Jianna Zhang.

**Writing – review & editing:** Zhi Wan, Yongli Gao.
